# *COX2* genetic variation, NSAIDs, and advanced prostate cancer risk

**DOI:** 10.1038/sj.bjc.6603874

**Published:** 2007-07-03

**Authors:** I Cheng, X Liu, S J Plummer, L M Krumroy, G Casey, J S Witte

**Affiliations:** 1 Department of Epidemiology and Biostatistics, University of California, San Francisco, CA 94143-0794, USA; 2 Center of Human Genetics, University of California, San Francisco, CA 94143-0794, USA; 3 Department of Pediatrics, Northwestern University, Feinberg Mary Ann and J Milburn Smith Child Health Research Program, School of Medicine and Children's Memorial Hospital and Children's Memorial Research Center, Chicago, IL 60614, USA; 4 Department of Cancer Biology, Lerner Research Institute, The Cleveland Clinic, Cleveland, OH 44195, USA

**Keywords:** COX2, polymorphism, NSAIDs, prostate cancer

## Abstract

Collective evidence suggests that cyclooxygenase 2 (COX2) plays a role in prostate cancer risk. Cyclooxygenase 2 is the major enzyme that converts arachidonic acid to prostaglandins, which are potent mediators of inflammation. Nonsteroidal anti-inflammatory drugs (NSAIDs) inhibit the enzymatic activity of COX2 and long-term use of NSAIDs appears to modestly lower the risk of prostate cancer. We investigated whether common genetic variation in *COX2* influences the risk of advanced prostate cancer. Nine single-nucleotide polymorphisms (SNPs) in *COX2* were genotyped among 1012 men in our case–control study of advanced prostate cancer. Gene–environment interactions between *COX2* polymorphisms and NSAID use were also evaluated. Information on NSAID use was obtained by questionnaire. Three SNPs demonstrated nominally statistically significant associations with prostate cancer risk, with the most compelling polymorphism (rs2745557) associated with a lower risk of disease (odds ratio (OR) GC *vs* GG=0.64; 95% confidence interval (CI): 0.49–0.84; *P*=0.002). We estimated through permutation analysis that a similarly strong result would occur by chance 2.7% of the time. Nonsteroidal anti-inflammatory drug use was associated with a lower risk of disease in comparison to no use (OR=0.67; 95% CI: 0.52–0.87). No significant statistical interaction between NSAID use and rs2745557 was observed (*P*=0.12). Our findings suggest that variation in *COX2* is associated with prostate cancer risk.

Cyclooxygenase 2 (COX2) is an inducible enzyme that converts arachidonic acid to prostaglandins, which are potent mediators of inflammation. Through the production of prostaglandins, COX2 is hypothesised to influence carcinogenesis by promoting cell proliferation, inhibiting apoptosis, stimulating angiogenesis, and mediating immune suppression (Kirschenbaum *et al*, 2001; Fujita *et al*, 2002; Nithipatikom *et al*, 2002; Wang *et al*, 2005). Multiple studies have shown increased expression of *COX2* in prostate tumours (Gupta *et al*, 2000; Kirschenbaum *et al*, 2000; Tanji *et al*, 2000; Yoshimura *et al*, 2000; Uotila *et al*, 2001), although one study reported overexpression not in tumour tissue but rather in proliferative inflammatory atrophy, a putative precursor lesion of prostate cancer (Zha *et al*, 2001). More recently, increased *COX2* expression has been correlated with higher tumour grade (Wang *et al*, 2005) and prostate cancer progression (Cohen *et al*, 2006). Nonsteroidal anti-inflammatory drugs (NSAIDs) inhibit the enzymatic activity of COX2, and inhibitors of COX2 suppress the growth of prostate cancer cells *in vitro* as well as prostate tumorigenesis *in vivo* (Liu *et al*, 2000; Gupta *et al*, 2004; Patel *et al*, 2005; Narayanan *et al*, 2006). Furthermore, long-term use of NSAIDs has been associated with a reduction in prostate cancer risk (OR=0.81; 95% CI: 0.70–0.94) among the American Cancer Society's Cancer Prevention Study II Nutrition Cohort, the largest prospective study to date of NSAIDs and prostate cancer having 5539 cases of prostate cancer (Jacobs *et al*, 2007).

Recently, two studies demonstrated that variants in *COX2* were associated with the risk of prostate cancer (Panguluri *et al*, 2004; Shahedi *et al*, 2006). In one study of African Americans, Nigerians, and European Americans, four promoter variants in *COX2* were evaluated and divergent patterns of association were observed across the three groups (Panguluri *et al*, 2004). Two variants, −1265 G/A (rs20415) and −899 G/C (rs20417), were associated with an increased risk of prostate cancer among African Americans, while the −297 C/G (rs5270) variant was associated with a reduced risk overall and among African Americans and European Americans (Panguluri *et al*, 2004). In a second study of a Swedish population, five *COX2* variants were examined and two variants, +3100 C/T (rs689470) and +8365 C/T (rs2043), were associated with a reduced risk of disease (Shahedi *et al*, 2006).

In light of the strong support for the involvement of *COX2* in prostate carcinogenesis coupled with the initial evidence that inherited differences in *COX2* may impact risk of disease, we evaluated the association between common genetic variation in *COX2* and prostate cancer risk in a case–control study of advanced prostate cancers. To date, this is the most comprehensive study of the genetic diversity of *COX2*. In addition, we investigated the possible interactive effects of *COX2* variation and NSAID use on prostate cancer risk.

## METHODS

### Study subjects

We recruited 506 advanced incident prostate cancer cases and 506 controls from the major medical institutions in Cleveland, Ohio (The Cleveland Clinic, University Hospitals of Cleveland, and their affiliates). Advanced prostate cancer cases were confirmed histologically and defined as having either a Gleason score ⩾7, or TNM stage ⩾T2c, or PSA at diagnosis >10 ng ml^−1^. Cases were contacted shortly following diagnosis (median time between diagnosis and recruitment=4.7 months). Restricting the cases to men diagnosed with advanced disease allows us to focus on the most clinically relevant prostate cancers. To help ensure that the controls were representative of the source population of the cases, controls were men who underwent standard annual medical exams at the collaborating medical institutions. Controls had no diagnosis of prostate cancer or any other non-skin cancers. All controls received a PSA test to detect occult prostate cancer. Controls were frequency matched to cases by age (within 5 years), ethnicity, and medical institution. Detailed information and descriptive characteristics for this case–control study has been reported previously (Liu *et al*, 2006).

Institutional Review Board approval was obtained from the participating medical institutions, and informed consent was obtained from all study participants.

### Tag SNP selection

We evaluated the genetic structure of *COX2* by using publicly available genotype data for European populations from the International HapMap project (www.hapmap.org) (Altshuler *et al*, 2005) and the Perlegen and Seattle SNP projects (National Heart, Lung, and Blood Institute Genome Variation Server; http://gvs.gs.washington.edu/GVS/). We assessed the common genetic variation of *COX2* (SNPs with minor allele frequencies (MAF) >5%) that spanned ∼2 kilobases (kb) upstream of the transcription start site and ∼1 kb downstream of the 3′ untranslated (UTR) region. For genetic characterization, seven SNPs (average density=1 SNP every 1.4 kb) and 14 SNPs (average density=1 SNP every 510 bp) were used from the HapMap and Perlegen/Seattle data, respectively. To thoroughly capture the common genetic variation across the locus, we utilised a pairwise tagging approach that reconstructed all SNPs across the locus (Carlson *et al*, 2004; de Bakker *et al*, 2005), using a criterion of a minimum pairwise *r*^2^⩾0.8 between the tag SNP and unmeasured SNP. The Tagger website (http://ww.broad.mit.edu/tagger) and Genome Variation Server (http://gvs.gs.washington.edu/GVS/) were used for tag SNP selection (Carlson *et al*, 2004; de Bakker *et al*, 2005). We did not attempt to capture the genetic variation of African populations because our sample size did not have sufficient power to conduct African American-specific analyses.

First using the HapMap data, we selected six tag SNPs (rs689466, rs2745557, rs2206593, rs4648307, rs4648261, and rs5272) to capture the common genetic variation across *COX2* (mean *r*^2^=1.0). Because genotyping assays for two SNPs (rs4648307, rs4648261) could not be designed and one SNP (rs5272) was monomorphic in our study population, we subsequently used the Perlegen and Seattle SNP data to maintain a comprehensive evaluation of the *COX2* locus. Using this data, we selected five tag SNPs (rs5277, rs2066826, rs5275, rs4648310, and rs689467) exclusive from the tag SNPs identified from HapMap. A genotype assay could not be designed for rs689467.

Two common SNPs, +8365 C/T (rs689470) and −899 G/C (rs20417), that were previously associated with prostate cancer (Panguluri *et al*, 2004; Shahedi *et al*, 2006) were also included as tag SNPs. In total, nine tag SNPs were selected for our case–control study: rs689466, rs20417, rs2745557, rs5277, rs2066826, rs5275, rs2206593, rs689470 and rs4648310.

### Genotyping

Genotyping was performed using the 5′ nuclease Taqman allelic discrimination assay using the manufacturer's predesigned primer/probe sets (rs5277, rs5275, rs2206593, rs689470) or custom-designed and manufactured primer/probe sets (rs689466, rs20417, rs2745557, rs2066826, rs4648310) (Applied Biosystems, Foster City, CA, USA). All assays were undertaken by individuals blinded to case–control status. For quality control, 2% replicate samples were included. The concordance rate for replicate samples was 100%. The average genotyping success rate was 99.9%. Further details of genotyping methods are described elsewhere (Liu *et al*, 2006). All SNPs were in Hardy–Weinberg equilibrium (at *P*>0.01 level).

### Nonsteroidal anti-inflammatory drugs

During an in-person computer-aided personal interview, men were asked about the amount and duration of previous aspirin and ibuprofen drug use (other types of NSAIDs were not assessed). We defined NSAID use as either aspirin or ibuprofen consumption at least twice a week for more than 1 month and no use as less than twice a week use of either of the medications for less than 1 month. For the dose/duration of use, we determined ‘pill-years’, which is the product of number of pills taken per day and years of drug use, for aspirin and ibuprofen. The summation of pill-years of aspirin use and ibuprofen use is the dose/duration of any NSAID consumption.

To investigate the hypothesis that genetic susceptibility to prostate cancer risk is associated with single causal variants, we evaluated the relationship between *COX2* genotypes and prostate cancer risk. Odds ratios (ORs) and 95% confidence intervals (CI) were estimated by unconditional logistic regression to examine the association between *COX2* SNPs and prostate cancer risk.

To potentially capture other unmeasured variants that may not be adequately captured by single markers, we evaluated the relationship between common *COX2* haplotypes and prostate risk. Haplotype frequencies among prostate cancer cases and controls were estimated by using genotype data of the tag SNPs as described by Stram *et al* (2003). Haplotype dosage (i.e. an estimate of the number of copies of haplotype h) for each individual and each haplotype, h, was computed using that individual's genotype data and haplotype frequency estimates were obtained from the E-M algorithm (Zaykin *et al*, 2002). Odds ratios and 95% CIs were then estimated by unconditional logistic regression for the association between *COX2* haplotypes and prostate cancer risk.

To examine the interaction between *COX2* genotypes and NSAID use, we dichotomized the latter into use and no use and assessed the effect of NSAID use stratified by *COX2* genotypes. To test for statistical interaction, the model contained separate terms for the main effect of *COX2* genotype, the main effect of NSAID use, and an interaction term for the cross product of *COX2* genotype and NSAID use. We also tested the interaction between *COX2* genotypes and dose/duration of NSAIDs (categorised into tertiles based on the distribution among controls).

All OR estimates were adjusted for age and medical institution and also for racial/ethnic group in any analysis that combined groups. All reported *P-*values are two-sided. Permutation testing was conducted to guide interpretation of nominally statistically significant SNP associations. Case–control status within strata of age (⩽62, 63–70, and >70 years), racial/ethnic group, and medical institution was randomly permuted 10 000 times for the nine *COX2* SNPs.

## RESULTS

First, we tested the association between *COX2* SNPs and prostate cancer risk in our study of 506 advanced cases and 506 controls. Three SNPs (rs2745557, rs2206593, and rs689470) were nominally statistically significantly associated with prostate cancer risk (Table 1). The strongest association was seen with rs2745557 (*P*=0.002); specifically, men carrying the GA genotype in comparison to men carrying the GG genotype had a significantly lower risk of disease (OR=0.64; 95% CI: 0.49–0.84). An overall consistent pattern of the GA genotype was seen across racial/ethnic groups (Table 1). Similarly, a lower risk of prostate cancer was observed for the CT genotype of rs2206593 (OR=0.58; 95% CI: 0.38–0.89); this polymorphism was moderated linked with rs2745557 (*D*′=1.00; *r*^2^=0.35). In contrast, a higher risk of disease was observed with the AA genotype of rs689470 in comparison to the GG genotype (OR=2.57; 95% CI: 1.17–5.64), with this SNP being more common among African Americans than Caucasians (MAF: 38 *vs* 2%, respectively). Overall, rs2745557 demonstrated the most compelling association with prostate cancer risk and its permutation *P-*value was 0.027, indicating that when permuting the data similar levels of significance were observed 2.7% of the time.

Next, we tested the association between *COX2* haplotypes and prostate cancer risk (Table 2). We identified one strong region of linkage disequilibrium (rs689466–rs5275) among Caucasians with six common haplotypes (>5%). A significant global haplotype effect for prostate cancer risk was observed (*P*=0.03). We observed a nominally significant association between the AGGGGA haplotype and prostate cancer risk (*P*=0.009). This haplotype could be defined by the G allele of rs5277, which was associated with risk among Caucasians (CG/GG *vs* CC: OR=1.36; 95% CI: 1.01–1.83).

We examined the potential interactive effects between the rs2745557 SNP and NSAIDs (Table 3). We previously reported an inverse association between NSAID use and prostate cancer risk in this study (OR=0.67; 95% CI: 0.52–0.87) (Liu *et al*, 2006). No significant statistical interaction was observed between rs2745557 and NSAID use (*P*=0.12). Among men carrying the GG genotype of rs2745557, a significant reduced risk of prostate cancer was observed with NSAID use (OR=0.58; 95% CI: 0.42–0.79). In contrast, among men carrying the GA/AA genotype of rs2745557, there was no significant effect with NSAID use. Similar patterns of associations were observed across racial/ethnic groups. No statistical interaction was observed between rs2745557 and dose/duration of NSAIDs (*P*=0.97).

## DISCUSSION

In this comprehensive evaluation of *COX2*, we identified a polymorphism (rs2745557) that was associated with a 36% reduction in risk of prostate cancer (*P*=0.002). In addition, the major G allele of this polymorphism in combination with NSAID use demonstrated an ∼40% lower risk of disease in comparison with the G allele and no NSAID use, while the minor A allele was associated with an ∼50% lower risk and was not influenced by NSAID use. However, a statistically significant interactive effect between rs2745557 and NSAID was not achieved. Our results suggest that inherited variation in *COX2* influences prostate cancer susceptibility.

In comparison with the previous study that detected an association between the −899 G/C (rs20417) variant and prostate cancer among African Americans (Panguluri *et al*, 2004), we did not detect any association with this SNP. Furthermore, while the previous Swedish study detected an inverse association between the +3100 C/T (rs689470) variant and prostate cancer (Shahedi *et al*, 2006), we observed no such association among Caucasians. However, we did observe a positive association with this variant among African Americans (Table 1). Reasons for these conflicting results are unclear and may be due to our limited power to detect rare effects (Caucasians: MAF=0.02 *vs* African Americans: MAF=0.38).

Our finding of an association between rs2745557 and prostate cancer risk was robust when subjected to permutation testing. Permutation testing empirically evaluates the robustness of nominally significant *P*-values and guides the interpretation of results when multiple hypotheses are tested (Hirschhorn and Daly, 2005). We determined that the genotype effect we observed would have occurred by chance less than ∼3% of the time. This demonstrates that *COX2* is a solid candidate gene for prostate cancer susceptibility and warrants further investigation.

Spurious association due to population stratification in our study is unlikely. For example, population stratification could occur if there is overrepresentation of African-American alleles among cases in comparison to controls. By matching cases and controls on racial/ethnic group as well as medical institution, the likelihood that population stratification may have biased our results is low. Moreover, we observed consistent effects among both African Americans and Caucasians, indicating that large-scale bias due to underlying population structure is unlikely to have occurred.

Our study has several limitations. Because of limited power to conduct African American-specific analyses, we did not characterise the common genetic variation of *COX2* among this population. Thus, we could not thoroughly assess the relationship between *COX2* variants and prostate cancer risk among African Americans. In addition, our study cannot exclude the possibility of an association between rare variants in *COX2* (MAF <5%) and prostate cancer risk.

The functional impact of rs2745557, an intronic variant, on COX2 activity is not yet known. It is possible that either this polymorphism itself or another linked marker may have biological effects on COX2 activity. We hypothesise that genetic variation in *COX2* may alter the production of inflammatory prostaglandins, which could ultimately influence prostate cancer susceptibility. Furthermore, environmental factors such as use of NSAIDs may modify the biological effects of *COX2* variation on disease risk. In particular, the combined effects of rs2745557 and NSAID use are of interest. Among men with the major allele of this polymorphism, those who were NSAID users had a slightly lower risk of prostate cancer than those who were non-NSAID users. While men with the minor allele of this polymorphism appeared to have no additional benefit of NSAID use, it is plausible that NSAID use may actively inhibit COX2 enzymatic activity only among those with the more prevalent major allele, while those with the variant allele may reach a threshold of COX2 inhibition such that NSAID use provides no added protection. To fully explore these potential interactive effects, larger well-powered studies are needed.

We previously reported that a functional variant (*LTA* C+80A) in lymphotoxin alpha, a proinflammatory cytokine, modified the protective effect of NSAIDs on prostate cancer (Liu *et al*, 2006). Specifically, a stronger inverse association between NSAIDs and prostate cancer was observed among men carrying the CC genotype (OR=0.43; 95% CI: 0.28–0.67). To further investigate the influence of the *LTA* C+80A variant on *COX2* and NSAID use, we examined the combined effects of rs2745557 and NSAIDs stratified by the CC and AC/AA genotypes for the *LTA* C+80A variant. Among men with the CC genotype for LTA C+80A, an 88% reduction in risk was observed among those with the AG/AA genotype for rs2745557 and who were also NSAIDs users (*P*<0.0001). Such findings highlight the complex nature of prostate cancer susceptibility, which is likely due to multiple susceptibility alleles acting in tandem with environmental components.

In this study, we demonstrate that common genetic variation in *COX2* influences the risk of prostate cancer. To date, this is the most comprehensive survey of common genetic variation at the *COX2* locus. Our finding supports previous reports showing an association between *COX2* variants and prostate cancer risk. Replication is critical in establishing an association between a variant and disease and additional studies either confirming or refuting these findings are needed. Furthermore, our study provides supporting evidence for the overall role of inflammation in prostate cancer susceptibility. By examining how inherited variation in inflammatory genes impacts risk of disease, we will further advance our understanding of prostate carcinogenesis and disease susceptibility.

## Figures and Tables

**Table 1:** Associations between *COX2* variants and prostate cancer risk

		**All groups^a^**			**African Americans^b^**			**Caucasians^b^**		
**SNP**		**Cases, *n* (%)**	**Controls, *n* (%)**	**OR (95% CI)**	**Cases, *n* (%)**	**Controls, *n* (%)**	**OR (95% CI)**	**Cases, *n* (%)**	**Controls, *n* (%)**	**OR (95% CI)**
rs689466	AA	337 (66.6)	357 (70.6)	1.00	67 (75.3)	77 (86.5)	1.00	270 (49.1)	280 (67.2)	1.00
	AG	154 (30.4)	134 (26.5)	1.22 (0.93–1.61)	20 (22.5)	12 (13.5)	1.94 (0.88–4.28)	134 (32.1)	122 (29.3)	1.14 (0.85–1.53)
	GG	15 (3.0)	15 (3.0)	1.06 (0.51–2.21)	2 (2.3)	0 (0)	—	13 (3.1)	15 (3.6)	0.90 (0.42–1.92)
										
rs20417^c^	GG	332 (65.7)	331 (65.5)	1.00	38 (42.7)	38 (43.2)	1.00	294 (70.7)	293 (70.3)	1.00
	GC	157 (31.1)	151 (29.9)	1.03 (0.78–1.36)	42 (47.2)	38 (43.2)	1.10 (0.59–2.07)	115 (27.6)	113 (27.1)	1.01 (0.75–1.38)
	CC	16 (3.2)	23 (4.6)	0.68 (0.35–1.33)	9 (10.1)	12 (13.6)	0.75 (0.28–1.99)	7 (1.7)	11 (2.6)	0.63 (0.24–1.66)
										
rs2745557	GG	364 (71.9)	318 (62.9)	1.00	69 (77.5)	56 (62.9)	1.00	295 (70.7)	262 (62.8)	1.00
	GA	126 (24.9)	172 (34.0)	0.64 (0.49–0.84)^e^	19 (21.4)	30 (33.7)	0.51 (0.26–1.01)	107 (25.7)	142 (34.1)	0.67 (0.50–0.90)^f^
	AA	16 (3.2)	16 (3.2)	0.87 (0.43–1.78)	1 (1.1)	3 (3.4)	0.27 (0.03–2.68)	15 (3.6)	13 (3.1)	1.02 (0.48–2.20)
										
rs5277	CC	359 (71.1)	384 (75.9)	1.00	86 (96.6)	83 (93.3)	1.00	273 (65.6)	301 (72.2)	1.00
	CG	134 (26.5)	116 (22.9)	1.25 (0.93–1.68)	3 (3.4)	6 (6.7)	0.48 (0.12–1.99)	131 (31.5)	110 (26.4)	1.32 (0.97–1.78)
	GG	12 (2.4)	6 (1.2)	2.18 (0.81–5.90)	0 (0)	0 (0)	—	12 (2.9)	6 (1.4)	2.21 (0.82–5.99)
										
rs2066826	AA	353 (69.8)	341 (67.5)	1.00	39 (43.8)	32 (36.4)	1.00	314 (75.3)	309 (74.1)	1.00
	AG	140 (27.7)	142 (28.1)	0.94 (0.70–1.25)	42 (47.2)	44 (50.0)	0.78 (0.42–1.47)	98 (23.5)	98 (23.5)	0.98 (0.71–1.36)
	GG	13 (2.6)	22 (4.4)	0.55 (0.27–1.13)	8 (9.0)	12 (13.6)	0.55 (0.20–1.50)	5 (1.2)	10 (2.4)	0.49 (0.17–1.46)
										
rs5275	AA	195 (38.6)	207 (40.9)	1.00	12 (13.5)	11 (12.4)	1.00	183 (44.0)	196 (47.0)	1.00
	AG	238 (47.1)	226 (44.7)	1.06 (0.70–1.58)	39 (43.8)	49 (55.1)	0.73 (0.29–1.85)	199 (47.8)	177 (42.5)	1.21 (0.91–1.60)
	GG	72 (14.3)	73 (14.4)	1.12 (0.86–1.47)	38 (42.7)	29 (32.6)	1.20 (0.46–3.14)	34 (8.2)	44 (10.6)	0.83 (0.51–1.35)
										
rs2206593^d^	CC	466 (92.1)	438 (86.7)	1.00	87 (97.8)	85 (96.6)	1.00	379 (90.9)	353 (84.7)	1.00
	CT	40 (7.9)	64 (12.7)	0.58 (0.38–0.89)^g^	2 (2.3)	3 (3.4)	0.65 (0.11–4.01)	38 (9.1)	61 (14.6)	0.58 (0.38–0.89)^h^
	TT	0 (0)	3 (0.6)	—	0 (0)	0 (0)	—	0 (0)	3 (0.7)	—
										
rs689470	GG	422 (83.6)	434 (85.9)	1.00	25 (28.1)	33 (37.5)	1.00	397 (95.4)	401 (96.2)	1.00
	GA	57 (11.3)	58 (11.5)	1.19 (0.73–1.93)	39 (43.8)	43 (48.9)	1.20 (0.61–2.37)	18 (4.3)	15 (3.6)	1.21 (0.60–2.43)
	AA	26 (5.2)	13 (2.6)	2.57 (1.17–5.64)^i^	25 (28.0)	12 (13.6)	2.79 (1.17–6.64)^j^	1 (0.2)	1 (0.2)	1.00 (0.06–16.21)
										
rs4648310	AA	473 (93.5)	485 (95.9)	1.00	88 (98.9)	87 (97.8)	1.00	385 (92.3)	398 (95.4)	1.00
	AG	30 (5.9)	20 (4.0)	1.54 (0.86–2.76)	1 (1.1)	2 (2.3)	0.49 (0.04–5.60)	29 (7.0)	18 (4.3)	1.67 (0.91–3.05)
	GG	3 (0.6)	1 (0.2)	3.10 (0.32–29.97)	0 (0)	0 (0)	—	3 (0.7)	1 (0.2)	3.12 (0.32–30.12)

a Adjusted for age, ethnicity, and institution.

b Adjusted for age and institution.

c rs20417=−899G/C.

d rs689470=+3100 C/T (reverse strand alleles).

e *P*=0.002;

f *P*=0.011;

g *P*=0.019;

h *P*=0.021;

i *P*=0.009;

j *P*=0.013.

**Table 2:** Associations between *COX2* haplotypes and prostate cancer risk among Caucasians^a^

**rs689466**	**rs20417**	**rs2745557**	**rs5277**	**rs2066826**	**rs5275**	**Haplotype % in cases/controls**	**OR (95% CI)**
A	G	A	C	G	A	16/20	1.00
G	G	G	C	G	A	19/18	1.29 (0.94–1.77)
A	G	G	C	G	G	17/16	1.30 (0.94–1.81)
A	G	G	G	G	A	19/15	1.56 (1.12–2.19)^b^
A	G	G	C	G	A	13/15	1.09 (0.78–1.53)
A	C	G	C	A	G	13/14	1.12 (0.79–1.59)

a Adjusted for age and institution.

b *P*=0.009.

**Table 3:** Odd ratios and 95% CIs for prostate cancer risk associated with *COX2* rs2745557 and NSAID use

		**rs2745557 GG**			**rs2745557 GA/AA**		
	**NSAID**	**Cases, *n* (%)**	**Controls, *n* (%)**	**OR (95% CI)**	**Cases, *n* (%)**	**Controls, *n* (%)**	**OR (95% CI)**
All groups^a^	No	264 (64.7)	144 (43.6)	1.00	64 (45.1)	80 (42.6)	1.00
	Yes	413 (61.0)	186 (56.4)	0.58 (0.42–0.79)^b^	78 (54.9)	108 (57.5)	0.86 (0.55–1.35)
African Americans^c^	No	44 (63.6)	24 (42.9)	1.00	14 (70.0)	22 (66.7)	1.00
	Yes	25 (36.2)	32 (57.1)	0.44 (0.21–0.91)^d^	6 (30.0)	11 (33.3)	0.73 (0.21–2.56)
Caucasians^c^	No	119 (40.5)	77 (29.8)	1.00	50 (41.0)	58 (37.4)	1.00
	Yes	175 (59.5)	181 (70.2)	0.62 (0.43–0.89)^e^	72 (59.0)	97 (62.6)	0.86 (0.53–1.40)

a Adjusted for age, ethnicity, and institution.

b *P*=0.0007.

c Adjusted for age and institution.

d *P*=0.0258;

e *P*=0.0089.
